# Microfluidic-assisted preparation of RGD-decorated nanoparticles: exploring integrin-facilitated uptake in cancer cell lines

**DOI:** 10.1038/s41598-020-71396-x

**Published:** 2020-09-02

**Authors:** Julio M. Rios De La Rosa, Alice Spadea, Roberto Donno, Enrique Lallana, Yu Lu, Sanyogitta Puri, Patrick Caswell, M. Jayne Lawrence, Marianne Ashford, Nicola Tirelli

**Affiliations:** 1 North West Centre for Advanced Drug Delivery (NoWCADD), School of Health Sciences, University of Manchester, Oxford Road, Manchester, M13 9PT UK; 2 Laboratory for Polymers and Biomaterials, Fondazione Istituto Italiano di Tecnologia, 16163 Genova, Italy; 3 Advanced Drug Delivery, Pharmaceutical Sciences, R & D, AstraZeneca, Cambridge, UK; 4 Wellcome Trust Centre for Cell-Matrix Research, Faculty of Biology, Medicine and Health, University of Manchester, Manchester Academic Health Science Centre, Manchester, M13 9PT UK; 5 Advanced Drug Delivery, Pharmaceutical Sciences, R & D, AstraZeneca, Macclesfield, UK; 6 Present Address: Cambridge Enterprise Limited, University of Cambridge, The Hauser Forum, 3 Charles Babbage Road, Cambridge, CB3 0GT UK

**Keywords:** Drug delivery, Targeted therapies

## Abstract

This study is about fine tuning the targeting capacity of peptide-decorated nanoparticles to discriminate between cells that express different integrin make-ups. Using microfluidic-assisted nanoprecipitation, we have prepared poly(lactic acid-co-glycolic acid) (PLGA) nanoparticles with a PEGylated surface decorated with two different arginine-glycine-aspartic acid (RGD) peptides: one is cyclic (RGDFC) and has specific affinity towards α_v_β_3_ integrin heterodimers; the other is linear (RGDSP) and is reported to bind equally α_v_β_3_ and α_5_β_1_. We have then evaluated the nanoparticle internalization in two cell lines with a markedly different integrin fingerprint: ovarian carcinoma A2780 (almost no α_v_β_3_, moderate in α_5_β_1_) and glioma U87MG (very high in α_v_β_3_, moderate/high in α_5_β_1_). As expected, particles with cyclic RGD were heavily internalized by U87MG (proportional to the peptide content and abrogated by anti-α_v_β_3_) but not by A2780 (same as PEGylated particles). The linear peptide, on the other hand, did not differentiate between the cell lines, and the uptake increase vs. control particles was never higher than 50%, indicating a possible low and unselective affinity for various integrins. The strong preference of U87MG for cyclic (vs. linear) peptide-decorated nanoparticles was shown in 2D culture and further demonstrated in spheroids. Our results demonstrate that targeting specific integrin make-ups is possible and may open the way to more precise treatment, but more efforts need to be devoted to a better understanding of the relation between RGD structure and their integrin-binding capacity.

## Introduction

Integrins are targetable receptors. They are transmembrane glycoproteins, which connect cell bodies to pericellular structures and therefore are critical in cell–cell and cell-environment interactions^1^. Structurally, they are heterodimeric proteins composed of non-covalently linked α and β subunits^2^. To date 18α and 8β subunits, and 24 different heterodimers have been reported in humans, with each heterodimer displaying unique binding properties, tissue distribution, and biological functions^1 ,3^. Although integrins are virtually ubiquitous, their heterodimeric identity and level of expression are a physio-pathological signature, which has a specific relevance in cancer^4^, e.g. in the formation of metastatic niches or in angiogenetic processes^5^. Most studies have focused either on integrins in peritumoral environments, e.g. on endothelial cells (ECs), critical to (neo)angiogenesis, or on those directly present on tumor cells^6^. It is now well known that α_v_-containing integrins are highly expressed in both scenarios, but poorly expressed on healthy cells^7^. In particular, α_v_β_3_ (e.g. in pro-tumoral ECs) is the most abundant integrin capable of regulating angiogenesis^8^. Among other heterodimers, also α_5_β_1_ has been clearly defined as a proangiogenic factor^9^. Interestingly, integrins are not only signalling and adhesion molecules overexpressed in pathological conditions (and therefore targetable); they are also efficient internalizers, involved in all major routes of endo/phagocytosis and rapidly recycled. It must be noted, however, that the mechanisms of internalization and recycling can be heterodimer-specific. For example, α_v_β_3_ integrin follows a rapid Rab4-dependent route from early endosomes to the plasma membrane, whilst α_5_β_1_ is trafficked back via Rab11-dependent recycling endosomes^10^. This multiple nature of pathology marker, cell surface binder and internalizer has led to the development of integrin-targeted therapies^11 ,12^. To this end, nanocarriers decorated with integrin ligands would enable an enhanced targeting efficiency via the combination of active targeting of integrin-overexpressing with passive, preferential (but not cell-specific) accumulation in tumoral masses, typical of colloids^13–15^. Indeed in vivo and ex vivo studies of arginine-glycine-aspartic acid (RGD) peptide-decorated polymeric nanoparticles have demonstrated a significant contribution of both active and passive accumulation mechanisms^16^. Numerous integrin ligands have been explored, e.g. by coating nanocarriers or polymers with the said recognition ligands or via the design of protein-drug conjugates^17^. Out of all the integrin-targeting ligands, RGD-containing peptides and their mimetics are the most popular^18^ and possibly the most versatile, also due to the availability of a variety of conjugation techniques^19^. Over the last decade, attention has specifically focused on peptides capable of targeting two of the most overexpressed heterodimers in cancer, i.e. α_v_β_3_ for glioblastoma or ovarian cancers^11 ,17,20^, and α_5_β_1_ for ovarian and lung cancers^17,21^, providing the means for the potential targeted delivery of classical chemotherapeutic agents^22–25^, nucleic acids^26,27^, proteins^28^, imaging agents^29,30^, or a combination of these^28,31,32^ to malignant cells.

It must also be noted that parameters such as the length, organization and sequence of the peptide, as well as the amount of exposed RGD and the mode of its presentation, can heavily affect the recognition and selectivity towards different integrins and therefore ultimately the targeting efficiency of the formulation^19^. To minimize these variables, in the present study we have adopted a microfluidics-based approach to the production of RGD-decorated and PEGylated polymer nanoparticles, which ensures the highest reproducibility both in nanoparticle dimension and surface coverage. We have previously produced particles via nanoprecipitation of poly(lactic acid-co-glycolic acid) (PLGA) (biocompatible, biodegradable, FDA-approved polymer^33^) from acetone into water in the presence of a PEGylated emulsifier (Pluronic F127)^34^. Herein, we have extended this approach to manufacture fluorescent PLGA nanoparticles with a controlled surface density of RGD peptides, which was obtained by using appropriate ratios of RGD end-functionalized Pluronic and the non-functional (OH-terminated) Pluronic. Further, we have used a cyclic (RGDc) and a linear (RGDl) peptide to produce particles with the same peptide content and exposure but different affinity for integrin heterodimers. We have then studied the nanoparticle uptake in two tumoral cell lines—A2780 (ovarian cancer) and U87MG (glioblastoma)—characterized by a considerably different integrin profile (here focusing on the α_v_β_3_/α_5_β_1_ difference). The study therefore tries to answer the question whether it is possible to obtain a selective cell uptake via matching integrin expression with the appropriate peptide(s) of the particle surface. We have done so by quantifying the uptake of a small library of nanoparticles (2 peptides, four surface densities each) and relating the integrin make-up of the two cell types, looking also at the mechanism of endocytosis and intracellular localization, and eventually confirming the results (for U87MG) on spheroids. It is probably necessary to add that, due to the size of this study, its results are difficult to extrapolate to other cell or other tumor types, therefore the conclusions we reach should be taken as a qualitative general point rather than a quantitative rule.

## Materials and methods

### Materials

RG502 poly(d,l-lactide-*co*-glycolide) (PLGA, Resomer) was obtained from Evonik (Wembley, UK). RG502 Resomer PLGA, acid terminated (PLGA-COOH), Pluronic F127, divinyl sulfone (DVS), QuantiPro BCA assay kit, phosphate buffered saline solution (PBS, 10 mM PO_4_^3−^, pH = 7.4, D8537), 4-(4,6-dimethoxy-1,3,5-triazin-2-yl)-4-methylmorpholinium chloride (DMT-MM) and 5-(N-Ethyl-N-isopropyl)amiloride (EIPA) were purchased from Sigma-Aldrich (Gillingham, UK). *d*6-dimethylsulfoxide (d6-DMSO) was supplied by VWR (Lutterworth, UK). Dichloromethane (DCM), acetonitrile (HPLC grade), tetrahydrofuran (THF) and Lissamine Rhodamine B ethylenediamine were from Fisher Scientific (Loughborough, UK). Sterile 2 mL round-bottom plastic tubes (#022363352) were acquired from Eppendor, UK. Arginine-glycine-aspartic acid (RGD) peptides: linear Ac-GCGYGRGDSPG-NH_2_ (purity > 99%, salt form: acetate salt) and cyclo(RGDFC) (purity > 99%, acetate content: 2.98%, TFA content: 0.43%) were obtained from Biomatik (Wilmington, USA). The human ovarian carcinoma A2780 (#93112519) and U87MG glioma (HTB-14) cell lines were acquired from Sigma-Aldrich (Gillingham, UK) and ATCC (Manassas, VA, USA), respectively. The RPMI 1640 (R0883) and MEM (51412C) cell culture media, Foetal Bovine Serum (FBS, F7524), L-Glutamine (G7513), Penicilin-Streptomycin (P4333) and Trypsin/EDTA (59417C) were all supplied by Sigma-Aldrich (Gillingham, UK). Costar polystyrene 12-well and 96-well plates with flat bottom, and 96-well ultra-low adherent (ULA) plates were bought from Corning, UK. The α_v_β_3_ (mAb 1976, clone LM604) and α_5_β_1_ (mAb 13) blocking antibodies were acquired from EMD Millipore, UK. The ibidi 8 well µ-Slides and LifeAct-TagGFP2 Protein were purchased from Ibidi GmbH (Germany). Green labelled-dextran, 70 kDa (#D7173), CellMask Green Plasma Membrane Stain (C37608), Hoechst 33342 (H1399) were all acquired from Thermo Fisher Scientific (Loughborough, UK). CellTiter 96 Aqueous One Solution Cell Proliferation Assay (MTS) was purchased from Promega (Southampton, UK).

### Preparation of Pluronic-α,[[ω]]-divinylsulfone (Pluronic-VS)

Pluronic F127 (20 g, 3.2 mmol of OH) was dissolved in toluene (100 mL) in a three neck round bottom flask connected to a Soxhlet (filled with dry 4 Å molecular sieves) and fitted with a reflux condenser, under dry argon atmosphere and dried by azeotropic distillation (4 h). The dried Pluronic F127 was cooled to 40 °C and NaH (15.36 mg, 0.64 mmol, corresponding to 1:0.2 Pluronic F127-OH/NaH molar ratio) added. The reaction was stirred at 40 °C for 40 min. The partially deprotonated Pluronic F127 was syphoned into another degassed reactor containing DVS (4.73 g, 40 mmol, corresponding to 80 mmol of double bonds, i.e. to 1:0.2:25 Pluronic F127-OH/NaH/double bonds molar ratio) previously dissolved in dry toluene (300 mL). The reaction was left stirring at 40 °C for 24 h. The mixture was filtered to remove any salt formed, neutralized by adding a drop of acetic acid, reduced in volume by rotary evaporation and precipitated twice in ice-cold diethyl ether. After precipitation, the polymer was washed three times with diethyl ether, dried under vacuum and stored at − 20 °C. Yield: 92% (based on the weight of recovered polymer/theoretical amount of polymer). Conversion: 63% (molar percentage of reacted OH groups), as confirmed by ^1^H NMR.

^1^H NMR was recorded on ca. 1% wt. polymer solutions in CDCl_3_, D_2_O or D_2_O/buffer mixture using a Bruker Avance 400 MHz spectrometer. The residual solvent signal was used as internal reference (ppm). NMR samples with a high content of non-deuterated aqueous solvent were analyzed using a pulse sequence with f1 presaturation for the selective suppression of the HOD signal.

**Figure 1:**
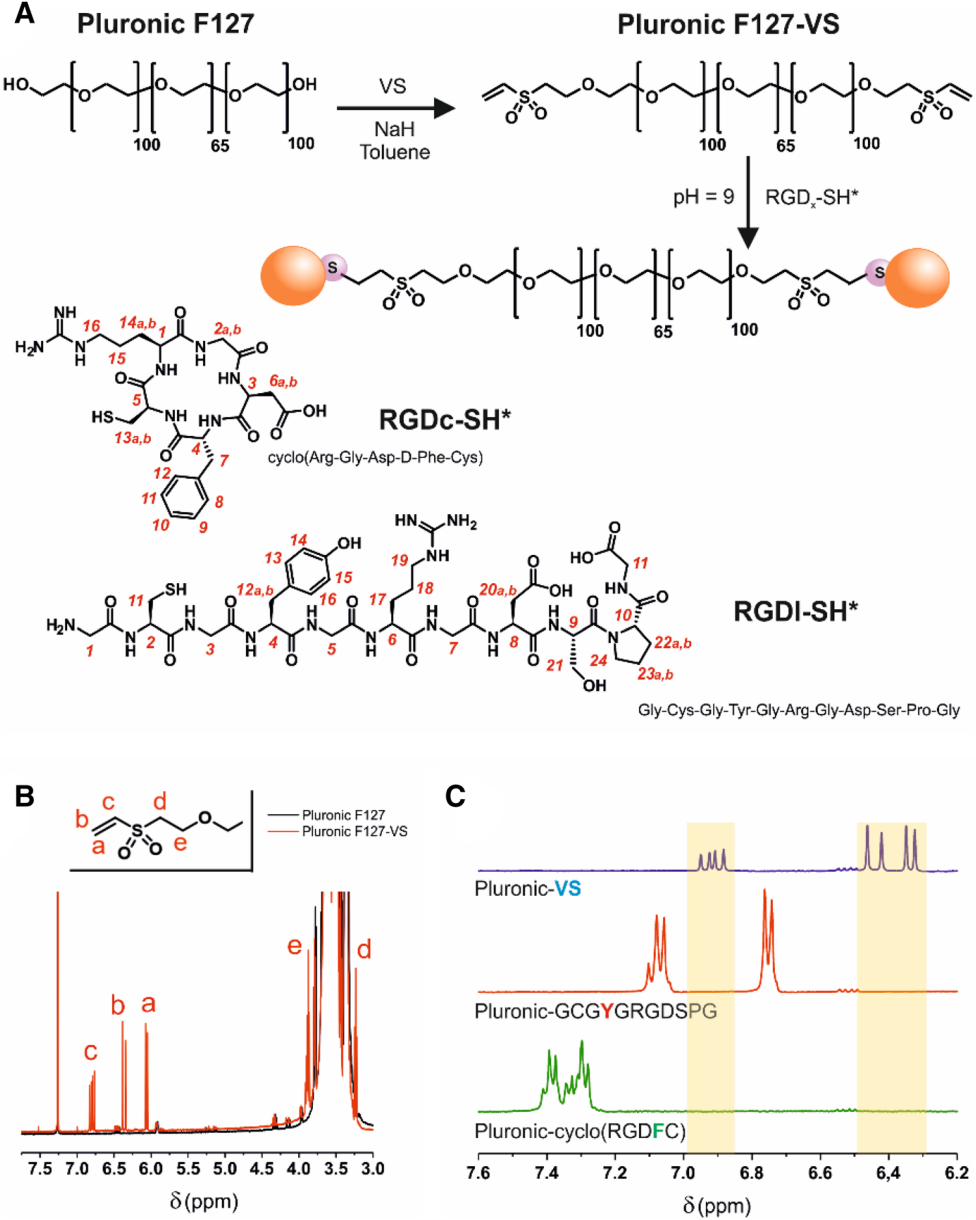
Pluronic F127-VS-RGD preparation and characterization. (**A**) Two-step reaction scheme and structure of the two thiol-containing RGD peptides. Numbers in red provide the assignment of the resonances in ^1^H NMR spectra (see experimental part). (**B**) ^1^H NMR spectra of Pluronic F127 (black) and Pluronic F127-VS (red) show the presence of vinylic unsaturations in PEG-VS. (**C**) Magnification of the ^1^H NMR spectra of Pluronic F127-VS and of its reaction products with either linear (RGDl) or cyclic (RGDc) peptide. The ^1^H NMR analysis confirmed complete conversion of Pluronic F127-VS: no trace of the vinyl groups (blue signals) was detected in the reaction mixture after a reaction time of 30 min. The red and green peaks are related to the presence of aromatic protons on the side chains of tyrosine (RGDl) and phenylalanine (RGDc), respectively.

*VS-Terminated Pluronic F127 (Pluronic-VS):* CDCl_3_, δ (ppm): 1.06–1.16 (m, methyl group in propylene oxide units, ≈ 311H); 3.23 (t, H**d**, 4H); 3.28–3.82 (m, methylene and methine groups in both ethylene and propylene units, ≈1721H); 3.87 (t, H**e**, 4H); 6.06 (d, H**a**, 2H); 6.37 (d, H**b**, 2H); 6.79 (dd, H**c**, 2H). For proton numbering, please refer to Fig. 1B.

### Preparation of Pluronic-VS-RGD conjugates

Peptide solutions were prepared in phosphate buffer (100 mM, pH = 9), previously degassed with argon, as follow: 21.5 mg and 11.6 mg of, respectively, linear and cyclic peptide (corresponding to ≈ 20 µmol of peptide) were dissolved in 1 mL of buffer. The pH was then adjusted to ≈ 9 using a 0.1 M NaOH solution and then both peptide solution volumes adjusted to 2.5 mL. Equal volumes of peptide solution and Pluronic F127-VS solution (10% w/v in phosphate buffer, pH = 9) were then mixed (30 min under stirring) in order to have a VS:peptide molar ratio of 1:2 (10 µmol of VS : 20 µmol of peptide. 100 mg of Pluronic = 8 µmol of Pluronic F127 and 16 µmol of OH end groups. The 63% of OH group, corresponding to 10 µmol of VS, were replaced with VS group). Finally, the compound was dialyzed for a minimum of 12 h against MilliQ water using a Spectra-Por Float-A-Lyzer G2 (MWCO: 3.5 KDa, Spectrum Labs), freeze dried and stored at − 20 °C until further use.

*Cyclo(RGDFC)* (*RGDc*), D_2_O, δ (ppm): 1.46–1.84 (m, H15 and H14a, 3H); 1.84–2.04 (m, H14b, 1H); 2.55 (dd, H6a, 1H), 2.67 (dd, H6b, 1H); 2.72 (dd, H13a, 1H); 2.78 (dd, H13b, 1H); 3.06 (dd, H7a, 1H); 3.13 (dd, H7b, 1H); 3.18–3.31 (m, H16, 2H), 3.52 (d, H2a, 1H); 4.21 (dd, H5, 1H); 4.25 (d, H2b, 1H); 4.43 (dd, H1, 1H); 4.67 (dd, H4, 1H); 4.71 (dd, H3, 1H); 7.26–7.47 (m, H8, H9, H10, H11 and H12, 5H). For proton numbering, please refer to Fig. 1A.

*Ac-GCGYGRGDSPG-NH*_*2*_ (*RGDl*), D_2_O, δ (ppm): 1.58–1.74 (m, H17 and H18, 4H); 1.74–1.88 (m, H23a, 1H); 1.88–2.18 (m, H22a and H23b, 2H); 2.07 (s, acetyl, 3H); 2.24–2.44 (m, H22b, 1H); 2.67 (dd, H20a, 1H); 2.73 (dd, H20b, 1H); 2.87–2.97 (m, H11, 2H); 2.99 (dd, H12a, 1H); 3.13 (dd, H12b, 1H); 3.24 (t; H19, 2H); 3.70–4.06 (m, H24, H21, H1, H3, H5, H7 and H11, 14H); 4.40 (dd, H6, 1H); 4.45 (dd, H10, 1H); 4.55 (dd, H2, 1H); 4.60 (dd, H4, 1H); 4.69 (dd, H8, 1H); 4.78 (dd, H9, 1H);6.87 (d, H14 and H15, 2H); 7.15 (d, H13 and H16, 2H). For proton numbering, please refer to Fig. 1A.

*Pluronic F127-RGDc*, D_2_O, δ (ppm): 1.20 (d, methyl group in propylene oxide units, ≈ 312H); 1.48–1.81 (m, H15 and H14a, 6H); 1.85–1.99 (m, H14b, 2H); 2.56 (dd, H6a, 2H); 2.68 (dd, H6b, 2H); 2.78–2.93 (m, H13, 4H); 3.06 (dd, H7a, 2H); 3.13 (dd, H7b, 2H); 3.17–3.31 (m, H16, 4H); 3.31–3.99 (m, H2a, H**a-e** (proximal to sulfone), methylene and methine groups in both ethylene and propylene units, ≈ 1802H); 4.01 (t, H20, 4H); 4.21–4.31 (m, H2b, H5, 6H); 4.42 (dd, H1, 2H); 4.67 (dd, H4, 2H); 4.71 (dd, H3, 2H); 7.27–7.49 (m, H8, H9, H10, H11 and H12, 10H). For proton numbering, please refer to Fig. 1A, B.

*Pluronic F127-RGDl*, D_2_O, δ (ppm): 1.20 (d, methyl group in propylene oxide units, ≈ 266H); 1.53–1.75 (m, H17 and H18, 8H); 1.75–1.88 (m, H23a, 2H); 1.88–2.18 (m, H22a and H23b, 4H); 2.07 (s, acetyl, 6H); 2.26–2.44 (m, H22b, 2H); 2.66 (dd, H20a, 2H); 2.71 (dd, H20b, 2H); 2.93–3.05 (m, H11, 4H); 3.09 (dd, H12b, 2H); 3.14 (dd, H12b, 2H); 3.25 (t, H19, 4H); 3.33–4.06 (m, H24, H21, H1, H3, H5, H7, H11, H**a-e** (proximal to sulfone), methylene and methine groups in both ethylene and propylene units, ≈ 1560H); 4.40 (dd, H6, 2H); 4.46 (dd, H10, 2H); 4.56–4.64 (m, H2 and H4, 4H); 4.68 (dd, H8, 2H); 4.75–4.79 (m, H9, 2H); 6.88 d (d, H14 and H15, 4H); 7.16 (d, H13 and H16, 4H). For proton numbering, please refer to Fig. 1A, B.

### Preparation of fluorescently labelled PLGA (PLGA-Rho)

PLGA-COOH was covalently conjugated to Lissamine Rhodamine B ethylenediamine to produce a fluorescently labeled PLGA (PLGA-Rho). All solutions were prepared in THF unless stated otherwise. Briefly, 50 mg of the polymer, PLGA-COOH (4.17 × 10^−3^ mmol of carboxylate) was dissolved in 10 mL by shaking overnight. After complete dissolution of the polymer, 2.5 mL of a solution containing 3.25 mg of rhodamine B (5.4 × 10^−3^ mmol, 1.3 eq.) was added to the polymer solution and stirred for 15 min, after which 2.5 mL of a solution containing 7 mg of DMT-MM (2.5 × 10^−2^ mmol, 6 eq.) was added. The reaction was stirred at 400 rpm at 25 °C for 72 h, and then quenched and precipitated using cold methanol. The polymer pellet was collected by centrifugation (10 min at 4,500 g), resuspended in dichloromethane, and precipitated again using cold methanol. The precipitate was then thoroughly washed with methanol and finally vacuum-dried (mass recovery: 80%). The degree of derivatization (0.8% mol) was determined by measuring the fluorescence intensity of the PLGA-Rho, using the rhodamine dye to relate the measured emission to the molar concentrations of the fluorophore (please note that this was later transformed in a molar ratio between functionalized and non-functionalized units in the polymer).

### Microfluidics-assisted nanoprecipitation

An automated microfluidic Asia 320 flow reactor (Syrris, Royston UK) was used for all nanoparticle preparations. Nanoparticles were prepared by mixing a 0.015%wt. aqueous surfactant solution (Pluronic F127 mixtures containing 0, 1, 10, 50 or 100% of RGD derivatives) with a 0.31%wt. PLGA (RG502) acetone solution using an Asia 1,000 μL 3-input reaction chip (Syrris part number: 2100146). For fluorescently labelled particles, the acetone solution contained a 10% wt. PLGA-Rho:PLGA mixtures. In all experiments acetone/water flow rate ratio of 0.2 and a total flow of 2 mL/min. Nanoparticles were left at 30 °C for 12 h under stirring in order to evaporate the acetone, filtered through a 0.22 µm PES membrane, divided in 1 mL aliquots and centrifuged in sterile 2 mL round-bottom plastic tubes. Particles were then resuspended in 1 mL of distilled water; five of these aliquots were combined and freeze dried to determine the dry weight and from there the mass recovery of the process. Typical mass recovery values: 90–95%. For cell culture purposes, the nanoparticles were further centrifuged and re-dispersed in culture media, typically at a concentration of 0.5 mg/mL.

### Nanoparticle characterization

#### Dynamic light scattering (DLS)

The Z-average (apparent hydrodynamic) size, polydispersity (PDI), count rates and ζ-potential of nanoparticle dispersions were obtained by DLS/electrophoretic mobility measurements at 25˚C using a Zetasizer Nano ZS (Malvern Instrument, UK). Samples were measured as prepared immediately after evaporating the organic phase.

#### Fluorimetry

A Synergy 2 multi-mode Biotek microplate reader (NorthStar Scientific Ltd, Leeds, UK) equipped with Gen5 software was used to characterize the fluorescence intensity of the nanoparticles prepared using different PLGA-Rho blends (excitation: 540/25 nm; emission: 620/40 nm; optical position: top 50%, light source: xenon flash).

#### Quantification of RGD nanoparticle coverages

The QuantiPro bicinchoninic acid (BCA) Assay Kit was used to assess the amount of RGD exposed at the surface of the RGD-functionalized PLGA nanoparticles. Briefly, nanoparticles (prepared from 0, 1, 10, 50 or 100% Pluronic F127-RGD mixtures) were centrifuged at 13,000 rpm for 15 min, concentrated 5 to 10 times (i.e. concentration range 5–10 mg/mL), and washed with distilled water (three steps of centrifugation/washings to ensure removal of unbound RGD-surfactant) before being incubated with the BCA solution overnight at 37 °C, following manufacturer recommendations for highest sensitivity. Prior to any measurements, each nanoparticle suspension was thoroughly centrifuged (at 13,000 rpm for 1 h at 4 °C) to eliminate any background contribution to the UV–Vis reading from light scattering of the particles. Finally, the amount of RGD peptide at the surface of the nanoparticles was estimated by using a calibration of soluble Pluronic F127-RGDx (linear or cyclic RGD peptides as appropriate) in water in the concentration range 0.12–125 µg/mL using a Synergy2 Biotek plate reader (excitation: 540/25 nm; emission: 620/40 nm) equipped with Gen5 software (sensitivity of the instrument adjusted to wells with the highest nanoparticle concentration of the calibration curve; optical position: top 50%; light source: xenon flash). Note that in this quantification we assumed an identical BCA assay response for both the Pluronic F127-RGD (“calibration curve standard”) and the RGD-functionalized nanoparticles (“sample”).

### Cell experiments

#### General cell culture

A2780 and U87MG cells were grown in MEM or RPMI 1640 respectively under standard conditions (5% (v/v) CO_2_, 37 °C), regularly tested for mycoplasma, and used at passage numbers below 20. The RPMI 1640 and MEM cell culture growth media were supplemented with 10% (v/v) FBS, 2 mM L-glutamine, and 1% (v/v) penicilin-streptomycin.

#### Nanoparticle uptake studies (flow cytometry)

U87MG and A2780 cells were seeded in Costar polystyrene 12-well plates with flat bottom at a density of 1.5 × 10^4^ and 3 × 10^4^ cells/cm^2^, respectively. Cells were left to adhere overnight and grow until a confluency of ~ 60 to 70% was reached. Cells were then exposed to 0.5 mg/mL rhodamine-labelled PLGA nanoparticles (centrifuged and redispersed in the appropriate cell culture medium) differing in (1) the format of RGD peptide exposed at their surface, i.e. either RGDc or RGDl, or (2) the amount of RGD peptide exposed at their surface, i.e. 1, 10, 50 or 100% Pluronic F127-RGD/–OH mixtures, for specific time points (4 , 8 and 16 h) in a humidified 5% (v/v) CO_2_ air atmosphere at 37 °C. A control of nontargeted particles, i.e. particles with exposed –OH end groups on their surface was also studied. Following a protocol previously described by our group^35^, the media were removed, the cells washed with PBS, detached by incubation with trypsin/EDTA (59417C, Sigma-Aldrich, UK) for 10 min at room temperature, and finally resuspended in 400 µL PBS per vial. The internalization of rhodamine labelled PLGA particles was determined on 10,000 live, individual cells with a BD LSRFortessa cytometer (BD Bioscience, San Jose CA, USA) equipped with FACSDiva software (v8.0.1). Data were analyzed with FlowJo (vX.0.7, Tree Star, Ashland, OR, USA) after gating single and live events in the FSC-A/FSC-H and FSC/SSC windows, respectively. Untreated cells were used as autofluorescence control in order to calculate any change in the median fluorescence intensity (MFI) over time, as well as the percentage of positive events for each cell line.

#### Selective inhibition of cell uptake pathways (U87MG cell line)

##### Flow cytometry

U87MG cells were plated in Costar polystyrene 12-well plates with flat bottom at a density of 1.5 × 10^4^ cells/cm^2^. Cells were left to attach and grow until a confluency of ~ 60 to 70% was reached. In order to block specific uptake pathways, cells were pre-treated for 30 min at 37 °C with growth medium containing either (1) EIPA 5 µM, (2) 5 µg/mL α_v_β_3_ antibody, (3) 5 µg/mL α_5_β_1_ antibody, (4) excess RGDc peptide 2.5 µM, (5) excess RGDl peptide 2.5 µM, or (6) PBS. Following pre-treatment and a thorough washing with PBS, the cells were exposed to rhodamine labelled PLGA particles (nontargeted or functionalized either with 100% RGDc or RGDl mixtures) in cell media containing the aforementioned inhibitors/competitors for 1 h at 37 °C. A control at 4 °C was also run to evaluate the effect of blocking the energy dependent processes on nanoparticle uptake. Following a previously described protocol^35^, the media were removed, the cells washed with PBS, detached by incubation with trypsin/EDTA (59417C, Sigma-Aldrich, UK) for 10 min at room temperature and finally resuspended in 400 µL PBS per vial. The internalization of rhodamine labelled nanoparticles was determined on 10,000 live, individual cells with a BD LSRFortessa cytometer (BD Bioscience, San Jose CA, USA) equipped with FACSDiva software (v8.0.1). Data were analyzed with FlowJo (vX.0.7, Tree Star, Ashland, OR, USA) after gating single and live events in the FSC-A/FSC-H and FSC/SSC windows, respectively. Untreated cells (negative control) were used during acquisition as autofluorescence control as well as to threshold the percentage of positive events for time point. Data were normalized to the positive control (cells pre-treated with PBS).

##### Confocal microscopy

U87MG cells were seeded with a density of 25,000 cells/cm^2^ in an Ibidi 8 well µ-Slide and let to attach overnight at 37 °C, 5% CO_2_. A volume of 200 µL/well of cell culture media containing 0.5 mg/mL of rhodamine-labelled particles (prepared from Pluronic F127-RGD 0% (i.e. nontargeted) or 100% RGDc or RGDl mixtures) were used to treat the cells for 1, 4 or 24 h. In order to analyze the endocytic pathway involved, media supplemented with 1 μg/mL Hoechst and 200 nM LysoTracker Green were added in each well for 15 min at 37 °C, 5% CO_2_. To analyze the macropinocytic pathway, cells were incubated with 0.25 mg/mL green labelled dextran 70 in media for 1 h at 37 °C, 5% CO_2_. After treatment, living cells were washed with PBS and immediately used for imaging using a confocal microscopy protocol of ours^36^. Confocal acquisitions were performed on live cells using a Leica TCS SP8 AOBS inverted confocal equipped with an immersion oil objective (63×/1.40/HCX PL Apo, Leica). For the acquisitions, the confocal settings were as follows: pinhole 1 airy unit, scan speed 400 Hz unidirectional, format 2048 × 2048 (1× zoom, pixel size 240 nm), format 1,024 × 1,024 (4× zoom, pixel size 89 nm). To eliminate any possible crosstalk between channels, images were collected with a sequential scan using the following laser lines and mirror settings: 405(100%) 410–483 nm; 488(25%) 495–550 nm; 561(100%) nm. Images were processed to enhance brightness using the ImageJ software (https://rsb.info.nih.gov/ij).

#### Nanoparticle penetration into U87MG multicellular spheroids

##### Flow cytometry

U87MG cells were seeded at a concentration of 1,000 cells (in 0.1 mL) in a 96-well ultra-low adherent (ULA) plate. The spheroids were grown at 37 °C, 5% CO_2_ for 10 days, replacing 50 μL of media every 3 days, until the spheroid mass reached a diameter of 400 μm. At day 10, 100 µL/well of cell culture media containing 1 mg/mL of rhodamine labeled particles (prepared from Pluronic F127-RGD 0% (i.e. nontargeted) or 100% RGDc or RGDl mixtures) was added to each well containing a spheroid to obtain a final nanoparticle concentration of 0.5 mg/mL in a total volume of 200 µL (at least 10 spheroids per experimental condition). After 24 h, the spheroids were washed with PBS and incubated with 1 μg/mL Hoechst 33,342 for 5 min at 37 °C, 5% CO_2_. Spheroids were then washed with PBS and incubated with trypsin/EDTA for 10 min at 37 °C, 5% CO_2_, further disaggregated with the help of a pipette and the fluorescence arising from Hoechst and rhodamine determined using flow cytometry. Untreated cells were used as negative control for autofluorescence and Hoechst fluorescence gradient was used to individuate two distinct regions: Hoechst positive cells (bright, Fig. 6A), considered to be at the edge of the spheroids, cells below the ones in the edge (middle, Fig. 6A), and Hoechst negative cells, which are considered as those at the core(dim, Fig. 6A). The internalization of rhodamine-labeled particles was determined on 10,000 live, individual cells using a BD LSRFortessa cytometer (BD Bioscience, San Jose CA, USA) equipped with FACSDiva software (v8.0.1). Data were analyzed with FlowJo (vX.0.7, Tree Star, Ashland, OR, USA) after gating single and live events in the FSC-A/FSC-H and FSC/SSC windows, respectively^35^.

The reason why larger spheroids (average diameter ≈400 µm) were used for flow cytometry vs confocal microscopy experiments is threefold: (1) a much higher cell number is needed to run flow experiments, (2) larger spheroids are easier to manipulate (i.e. to see and handle) in experiments involving multiple passages, and more importantly (3) to obtain a well-defined Hoechst gradient. A quick incubation with Hoechst (for 10 min) is enough to create a three-region gradient in 400 µm spheroids; smaller spheroids would encumber this operation as Hoechst would quickly penetrate from the surface down to the core.

##### Confocal microscopy

Spheroids with diameter of about 250 μm were incubated with a final nanoparticle concentration of 0.5 mg/mL in cell media (0.2 mL/well). After a 24 h incubation, the spheroids were washed with PBS and incubated with 1 μg/mL Hoechst 33,342 and 5 μg/mL CellMask for 5 min at 37 °C, 5% CO_2_. The spheroids were then washed and used for imaging using a confocal microscope. Confocal acquisitions were performed on live cells using a Leica TCS SP8 AOBS inverted confocal equipped with an immersion oil objective (20×/1.40/HCX PL Apo, Leica). For the acquisitions, the confocal settings^36^, were as follows: pinhole 1 airy unit, scan speed 400 Hz unidirectional, format 2048 × 2048 (pixel size 280 nm). To eliminate any possible crosstalk between channels, images were collected with a sequential scan using the following laser lines and mirror settings: 405(100%) 410–483 nm; 488(25%) 495–550 nm; 561(100%) nm. Images were processed to enhance brightness using the ImageJ software (https://rsb.info.nih.gov/ij).

For the avoidance of doubt, we decided not to use spheroids larger than 250 µm in diameter for microscopy studies (as opposed to the 400 µm spheroids used for flow cytometry) since the transmitted light decreases with spheroid size. The use of 250 µm spheroids turned out to be the best compromise between ease of handling and light transmission at the core of the sphere.

## Results and discussion

### Preparation of Pluronic-VS and conjugation of RGD peptides

We recently developed a robust and highly reproducible method to prepare PEGylated nanoparticles via a continuous-flow microfluidic approach^34^. In brief, the manufacturing procedure consisted of mixing an acetone solution of PLGA with Pluronic F127 in water; the precipitation in the form of Pluronic-stabilized PLGA nanoparticles is performed in a cross-shaped microfluidic chip (160 μm-wide capillaries), which provides a flow-focused, very reproducible mixing fluidodynamics. Pluronic F127 was used in its commercially available, OH-terminated form, or after the introduction of peptides as terminal groups. To this end, we have used a two-step reaction sequence based on the introduction of Michael acceptor groups at the ends of the Pluronic terminal PEG blocks, and their successive Michael-type addition with appropriate thiol-containing peptides (Fig. 1A). Firstly, the OH groups of Pluronic F127 were reacted with an excess of the bifunctional vinyl sulfone (VS) in the presence of catalytic amounts of NaH, to form Pluronic F127-VS. The reaction conditions, derived from a previously published VS-based PEGylation protocol^37^, rendered a derivatization yield of 63 mol% of the free OH groups (Fig. 1B). The VS group presents a remarkable reactivity and selectivity towards thiols, making bioconjugation reactions possible with high yield, high purity and in one step^38^, as we have recently demonstrated for the functionalization of PEG-VS with cysteine-containing fibrinopeptides^39^. In the past, we have also shown that the Michael-type addition of thiol-containing peptides is governed by the cysteine pKa of a cysteine (thiol^40^) and we have used this reaction e.g. to introduce cysteine-bearing RGD sequences e.g. on hyaluronic acid^41^.

In the current work, we conjugated Pluronic F127 with one of two different peptides, namely (1) a fibronectin-derived linear RGD peptide (RGDl, GCGYGRGDSPG), targeting both α_v_β_3_ and α_5_β_1_ heterodimers^42,43^, and possibly some others, with typically low affinity values (IC_50_ > 100 nM^44^), and (2) a cyclic RGD peptide (RGDc, sequence: RGDFC), which exclusively targets α_v_β_3_ dimers with a high binding affinity (IC_50_ = 12 nM/K_i_ = 41 ± 0.5 nM for the soluble α_v_β_3_ receptor^45^). In our hands, we have achieved a quantitative conversion of VS groups with both RGD peptides as judged by the complete disappearance of their characteristic ^1^H NMR resonances after only 30 min reaction with both RGD peptides (Fig. 1C). The success of the conjugation of the RGD peptides to Pluronic F127-VS was further supported by the presence, in the ^1^H NMR spectra, of aromatic protons from the side chains of tyrosine (Y) and phenylalanine (F), arising from the RGDl and RGDc structures, respectively (bottom two spectra in Fig. 1C).

### Preparation and characterization of nanoparticles

We have prepared PLGA nanoparticles using a continuous-flow microfluidic system (Asia 320 from Syrris Ltd); this automated system allowed to easily and reproducibly obtain a library of particles with identical size, bulk composition and PEGylated surface, but differing both in the identity and in the amount of the decorating peptide (RGDc or RGDl).

The nanoprecipitation conditions used herein were analogous to our previously optimized protocol that rendered Pluronic F127/PLGA particles of a desired size, low polydispersity, high stability, good surface coverage and a spherical shape^34^. For the preparation of nanoparticle formulations exposing varying densities of peptide, we used RGD-functionalized and pristine Pluronic F127 mixed in different ratios as surfactants in the nanoprecipitation experiments. This led to formulations with a 0, 1, 10, 50 or 100% of Pluronic F127-RGD/–OH ratio (assuming the two surfactants to have the same relative ratio on the particle surfaces as in the feed).

**Figure 2:**
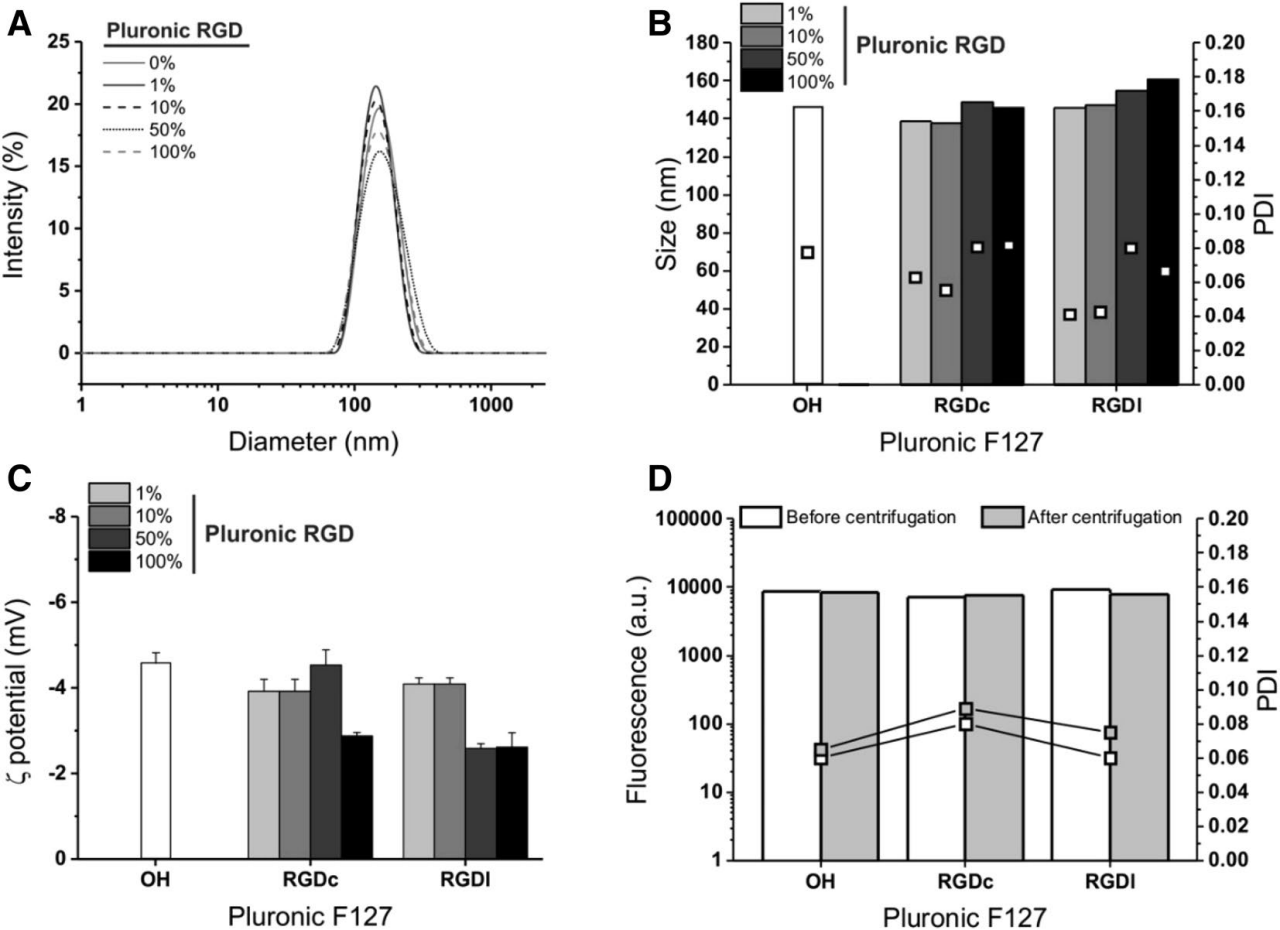
Physico-chemical characterization of PLGA nanoparticles as a function of format and concentration of Pluronic F127-RGD. The intensity size distribution (**A**, shown only for the linear RGD peptide) and the apparent hydrodynamic size and polydispersity (**B**) values show that the presence of Pluronic F127-RGD did not substantially alter the nanoparticle dimensions, with only slight changes in their **ζ** potential (**C**); the data for the latter are measured in 10 mM NaCl, for values in deionized water see Table 1. *n* = 3. (**D**) The fluorescence of the nanoparticles remains unchanged after centrifugation regardless of the surfactant format (100% Pluronic F127-RGD/–OH mixtures for both RGDc and RGDl) (*n* = 3).

We have evaluated the influence of the nature and density of the RGD ligand expressed on the surfactant of the nanoparticles on their apparent hydrodynamic size, polydispersity (PDI) and **ζ** potential (see Fig. 2A–C; Table 1). Particles of around 145–160 nm in diameter with remarkably low polydispersity values (< 0.1 in all cases) were obtained. These findings replicate previous results of our group for the PLGA/Pluronic F127 nanoprecipitation in cross-shaped chips^34^. Of note, particle size and dispersity can be affected by both microfluidic conditions and macromolecular parameters^46^. In general, neither the format nor the density of the peptide altered much the characteristics of the nanoparticles, albeit with a small size increase with 50% RGDl/OH and with 100% RGDc or RGDl surfactant mixtures. This is possibly due to a slightly lower capacity of steric stabilization offered by surfactants that bear ionic/ionizable or hydrogen donor groups in addition to PEG.

**Table 1:** Physico-chemical characterization of PLGA nanoparticles prepared via nanoprecipitation from 1, 10, 50 or 100% Pluronic F127-RGD/–OH mixtures.

F127-RGDx/F127-OH (wt%)	Z-average size (nm)	PDI	ζ-potential (mV)		F127-RGDx^a^ (µg/mg particle)	No. of RGD per particle^b^ (exp./theor.)
			ddH_2_O	10 mM NaCl		
**F127-OH**						
0%	145 ± 3	0.07 ± 0.03	− 30 ± 3	− 4.6 ± 0.2	n.d	n.d
**F127-RGDc**						
1%	138 ± 2	0.06 ± 0.03	− 27 ± 2	− 3.9 ± 0.3	n.d	n.d./148
10%	138 ± 4	0.05 ± 0.03	− 29 ± 6	− 3.9 ± 0.3	n.d	n.d./1,480
50%	148 ± 1	0.09 ± 0.02	− 35 ± 5	− 4.5 ± 0.4	30 ± 5	2,321 ± 500/7,400
100%	145 ± 2	0.09 ± 0.02	− 29 ± 2	− 2.9 ± 0.1	47 ± 2	4,643 ± 200/14,800
**F127-RGDl**						
1%	145 ± 2	0.04 ± 0.01	− 28 ± 4	− 4 ± 0.1	n.d	n.d./148
10%	147 ± 2	0.03 ± 0.01	− 33 ± 3	− 4 ± 0.1	10 ± 2	427 ± 190/1,480
50%	154 ± 5	0.08 ± 0.02	− 26 ± 4	− 2.6 ± 0.1	28 ± 4	2,134 ± 380/7,400
100%	160 ± 2	0.06 ± 0.02	− 27 ± 2	− 2.6 ± 0.3	45 ± 8	4,269 ± 760/14,800

*n.d.* non-detectable.

^a^The amount of F127-RGDx was measured by BCA assay after centrifugation and removal of unbound Pluronic F127-RGD and concentration of particles (×5–×10) by centrifugation, employing the appropriate Pluronic F127-RGDx standards. *n* = 3 independent measurements.

^b^The experimental values are calculated from the BCA readouts assuming a particle density of 1.24 g/cm^3^ (value for PLGA^47^) and molecular weights of around 13,350 and 13,900 g/mol respectively for Pluronic F127-RGDc and for F127-RGDl.

In comparison to other PEGylated PLGA nanoparticle systems in the literature, our apparent hydrodynamic sizes are in line with those reported for RGD/RGD peptidomimetic-functionalized nanoparticles (preferential delivery of paclitaxel to HUVEC endothelial cells)^24^, of around 130–150 nm, and considerably smaller than those reported for RGDc-decorated ones (targeted delivery of doxorubicin to cancer cell lines)^22^, of around 400 nm. In respect of surface charge all particles exhibited rather high negative **ζ**-potential values in deionized water, with or without peptide (Table 1), which is rather common for aliphatic polyester nanoparticles due to terminal carboxylic groups. However, **ζ** potential values dropped close to zero in 10 mM NaCl (Fig. 2C), which suggests a low likelihood of ionic interactions and therefore also of protein adsorption in biological fluids.

A note on particle dimensions: we report values calculated from the DLS intensity distribution; we have previously shown^34^ that in volume distributions obtained through field flow fractionation, the size of these particles is slightly higher than 100 nm, and when dried for TEM analysis they look even smaller (see Supplementary Fig. 1SI), due to dehydration of the surface PEG chains.

The preparative conditions employed here had previously been optimized to minimize the presence of free surfactant^34^. It is worth pointing out that any free, integrin-targeting surfactant (possibly competing with the RGD-decorated nanoparticles) is assumed to be removed via three rounds of centrifugation/resuspension, which at the same time allowed to recover most of the particle mass (typically 90–95% as estimated via DLS count rates^48^). It is possible to have a (rough) estimate of the density of RGD residues based on the literature values of Pluronic F127 interfacial area, and assuming that the peptides do not affect it. According to a study of Alexandridis, the Pluronic F127 surface area may range from 4.3 nm^2^ per molecule when adsorbed on carbon black (from DLS measurements) to 6.8 nm^2^ when unconstrained in micelles (calculated from the overall micelle radius and the average number of association from SANS measurements at 50 °C)^49^; since our situation is possibly more similar to micelles—carbon black is massively more hydrophobic—we have considered a value of about 6 nm^2^/molecule. Assuming particles to have a spherical shape and a size of about 150 nm, i.e. an average surface area of ~ 70,600 nm^2^, this would equate to ~ 11,800 Pluronic F127 molecules per particle, corresponding to about 14,800 peptides per particle (bear in mind the 63% end functionalization of Pluronic F127). Experimentally, the amount of exposed peptides was measured via the BCA assay (Table 1, last column from the right), assuming nanoparticles to have the same density as bulk PLGA; the experimental peptide surface density scaled with the amount of RGD-bearing Pluronic F127 and was in the same order of magnitude as the theoretical prediction, but significantly lower than that. We ascribe the discrepancy essentially to the difficult accessibility of most of the peptides on the nanoparticle surfaces.

### Evaluation of nanoparticle uptake and intracellular sorting

As the next step, we evaluated two experimental approaches to obtain traceable particles for use in our biological experiments. The first one consisted of the physical entrapment of small hydrophobic fluorophores (DiL^50^ or Nile Red^51^) co-dissolved with PLGA during nanoprecipitation. We recorded macroscopic agglomeration using DiL (i.e. 2 and 20 µg/mL; see Supplementary Information, Fig. 2SI), which was therefore abandoned. On the contrary, Nile Red up to 50 µg/mL in the polymer solution did not compromise particle stability and returned brightly fluorescent nanoparticles (Supplementary Fig. 3SI), but in preliminary in vitro experiments rapidly (< 1 min) leaked and stained perinuclear regions of A2780 cells (Supplementary Fig. 4SI). Although this phenomenon is often neglected in literature, it has been previously reported that a rapid increase in intracellular fluorescence is caused by Nile Red leaking out of the particles and associating, due to its lipophilic nature, with serum proteins, thereby leading to an overestimation of nanoparticle uptake^52^.

**Figure 3:**
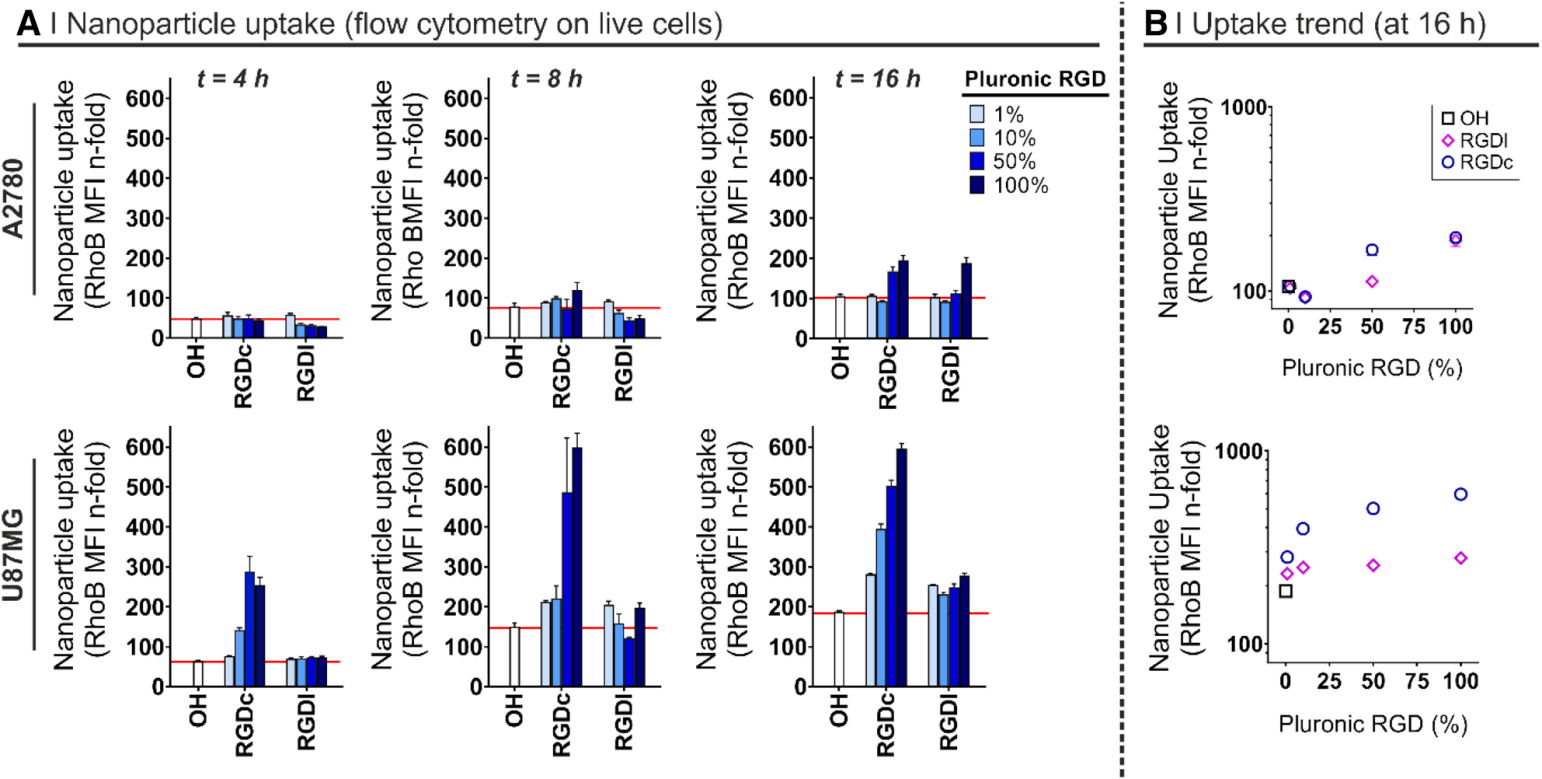
(**A**) Nanoparticle uptake after a 4, 8 or 16 h incubation in cell culture media as a function of the RGD format and density on the nanoparticle surface by A2780 ovarian carcinoma cells (top) and U87MG glioma cells (bottom). The nanoparticle uptake was followed via flow cytometry by monitoring the fluorescence of the nanoparticle hydrophobic core, i.e. rhodamine B-labelled PLGA (PLGA-Rho). Non-targeted nanoparticles (i.e. prepared with Pluronic F127-OH) were used as control of non-specific uptake (n = 3). Please note the red line is intended to help the reader identify the uptake of non-targeted particles. (**B**) Cross-correlation between the Pluronic F127-RGD mixture used to prepare nanoparticles and the nanoparticle uptake (flow cytometry on live cells at 16 h). RGDc = cyclic RGD peptide, specifically targeting α_v_β_3_ heterodimers. RGDl = linear RGD peptide, in principle binding to both α_v_β_3_ and α_5_β_1_ heterodimers.

**Figure 4:**
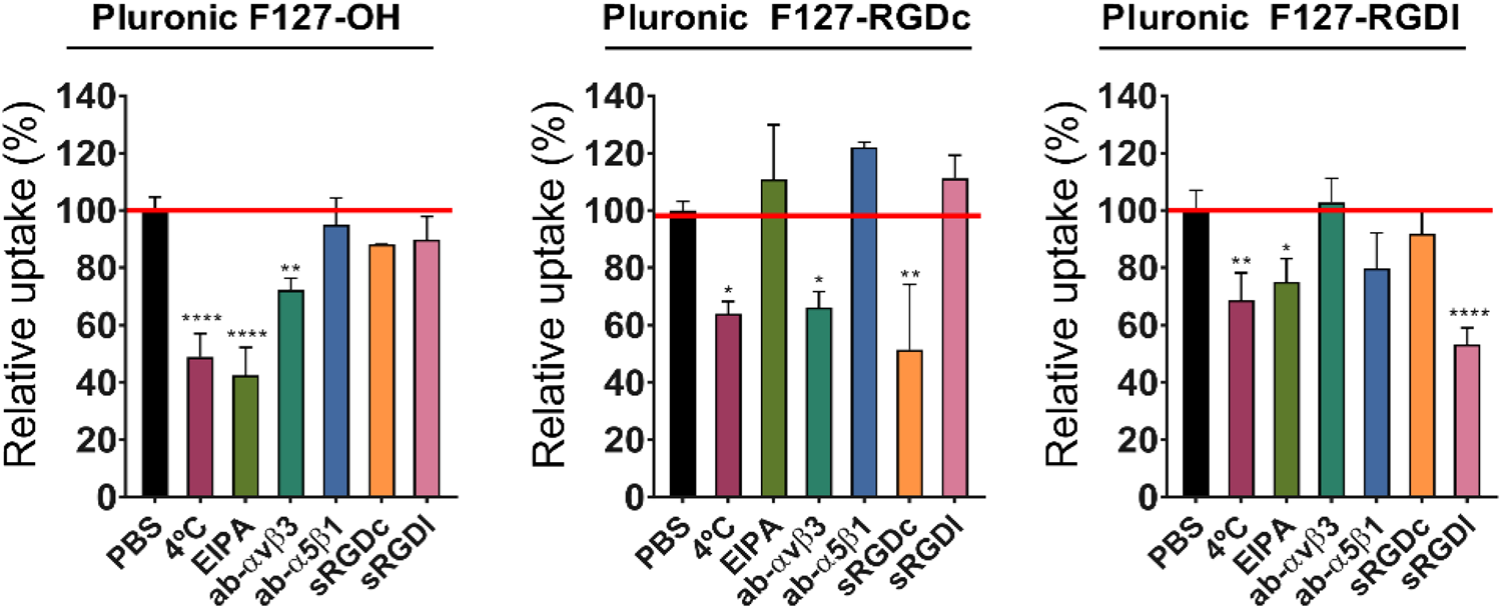
Uptake mechanism in U87MG cells as a function of RGD format on the nanoparticle surface. Nanoparticles were prepared from PLGA-Rho in single-emulsifier experiments (i.e. 100% F127-OH or 100% F127-RGDx); their uptake was measured by flow cytometry on live cells after pre-treatment for 30 min and co-incubation with different agents and nanoparticles for 1 h at 37 °C, and expressed in relation to the control experiment performed without inhibitors (i.e. PBS-treated cells). Please note that all cells showed evidence of internalization (see Supplementary Fig. 6SI.B), they just differed in the amount of uptake. RGDc = cyclic RGD peptide, specifically targeting α_v_β_3_ heterodimers. RGDl = linear RGD peptide, binding to both α_v_β_3_ and α_5_β_1_ heterodimers. Statistical analysis (One-way ANOVA), n = 3.

As an alternative, rhodamine B was directly conjugated to a carboxylic-terminated PLGA to yield a polymer-dye conjugate (i.e. PLGA-Rho) that would only be found in the nanoparticles, completely excluding the possibility of leakage. We used mixtures containing 10, 20 and 50% wt. of PLGA-Rho in unlabeled PLGA (in the input acetone solution), obtaining particles with unaltered physico-chemical characteristics (Supplementary Fig. 5SI,A–C). A 10% PLGA-Rho/PLGA ratio was sufficient to yield bright and easily detectable nanoparticles (unaffected by either the presence of RGD on the surface of particles, their centrifugation or filtration, see Fig. 2D and Supplementary Fig. 5SI,D), and this ratio was then employed for all further cell uptake experiments, followed via either confocal microscopy or flow cytometry. For these studies, we employed the glioma U87MG and the ovarian carcinoma A2780 cell lines, which express integrin heterodimers differing both in amount and type^53^: the former have very high levels of α_v_β_3_ integrins (~ 2.25 × 10^5^ surface receptors/cell) and still high levels of α_5_β_1_ integrins (~ 1.25 × 10^5^ surface receptors/cell), the latter minimal levels of α_v_β_3_ and moderate levels of α_5_β_1_ integrins (about half of what reported for U87MG). Therefore, the α_v_β_3_ integrin selective RGDc is expected to strongly favor uptake in U87MG (due to their high α_v_β_3_ expression) over A2780; the rather α_v_β_3_/α_5_β_1_ unselective RGDl should be conducive to a rather similar uptake in the two cell lines.

**Figure 5:**
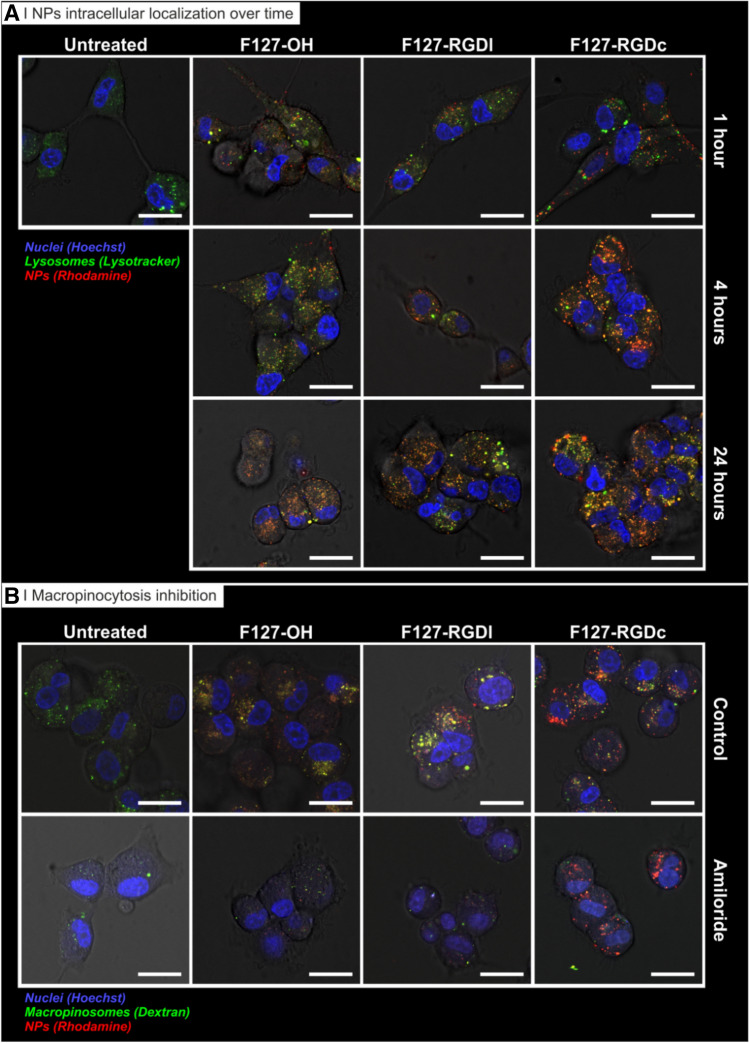
Uptake of rhodamine-labelled nanoparticles (red) prepared from Pluronic F127-OH, Pluronic F127-RGDl or Pluronic F127-RGDc (0 and 100% RGD/OH mixtures) by U87MG glioblastoma cells (confocal microscopy on live cells). (**A**) Nanoparticle intracellular localization over time. Cells were treated for 1 h with the three different nanoparticle formulations in cell culture media (0.5 mg/mL) at 37˚C. Nuclei: Hoechst stain (blue); lysosomes: Lysotracker (green). Scale bar = 20 μm. (**B**) Macropinocytosis inhibition. Cells were treated for 1 h with the three nanoparticle formulations in cell culture media (0.5 mg/mL) at 37˚C with or without a pre-treatment with 5 µM of EIPA for 30 min. Nuclei: Hoechst stain (blue); Macropinosomes: Dextran (green). Scale bar = 20 μm.

The 4, 8 and 16 h incubation of cell monolayers with rhodamine-labeled nanoparticles (Fig. 3) showed that:

A. Non-targeted particles had a slow uptake in the two cell lines, slightly more rapid in U87MG.
B. The internalization in A2780 increased when 50% or more Pluronic F127 expressed the RGD peptides, but only at 16 h and with no effect of the peptide format.
C. the uptake was significantly enhanced (namely a 2–6 fold increase) in U87MG at any given time point, but only with the α_v_β_3_ integrin selective RGDc, and increasingly with its increasing density.
D. RGDl only marginally improved nanoparticle uptake in comparison to non-targeted controls; this may indicate that this Pluronic F127 bound peptide has a low affinity for both integrins, or low for α_5_β_1_ and almost zero for α_v_β_3_ (although literature seems to exclude this possibility^54^). We can further speculate that RGDl may also promote carrier exocytosis, or that it may not induce integrin clustering, which can account for improved (high avidity) binding to nanoparticles^2^.

In view of the uptake results, we have selected the U87MG cell line as the most suitable model for further experiments, starting with the confirmation that their nanoparticle uptake was in fact mediated by a specific, integrin mediated pathway. To this end, cells were pre-incubated for 30 min with several uptake inhibitors or competitors, including excess soluble RGD peptides^55,56^ or antibodies against specific heterodimers^57^, followed by a 1 h co-incubation with rhodamine-labelled particles (please refer to Supplementary Information 4SI and Supplementary Fig. 6SI.A for cytotoxicity studies of these inhibitors/competitors). The intracellular fluorescence was then analyzed via flow cytometry, using cells pre-treated with PBS as positive uptake control (Fig. 4).

**Figure 6:**
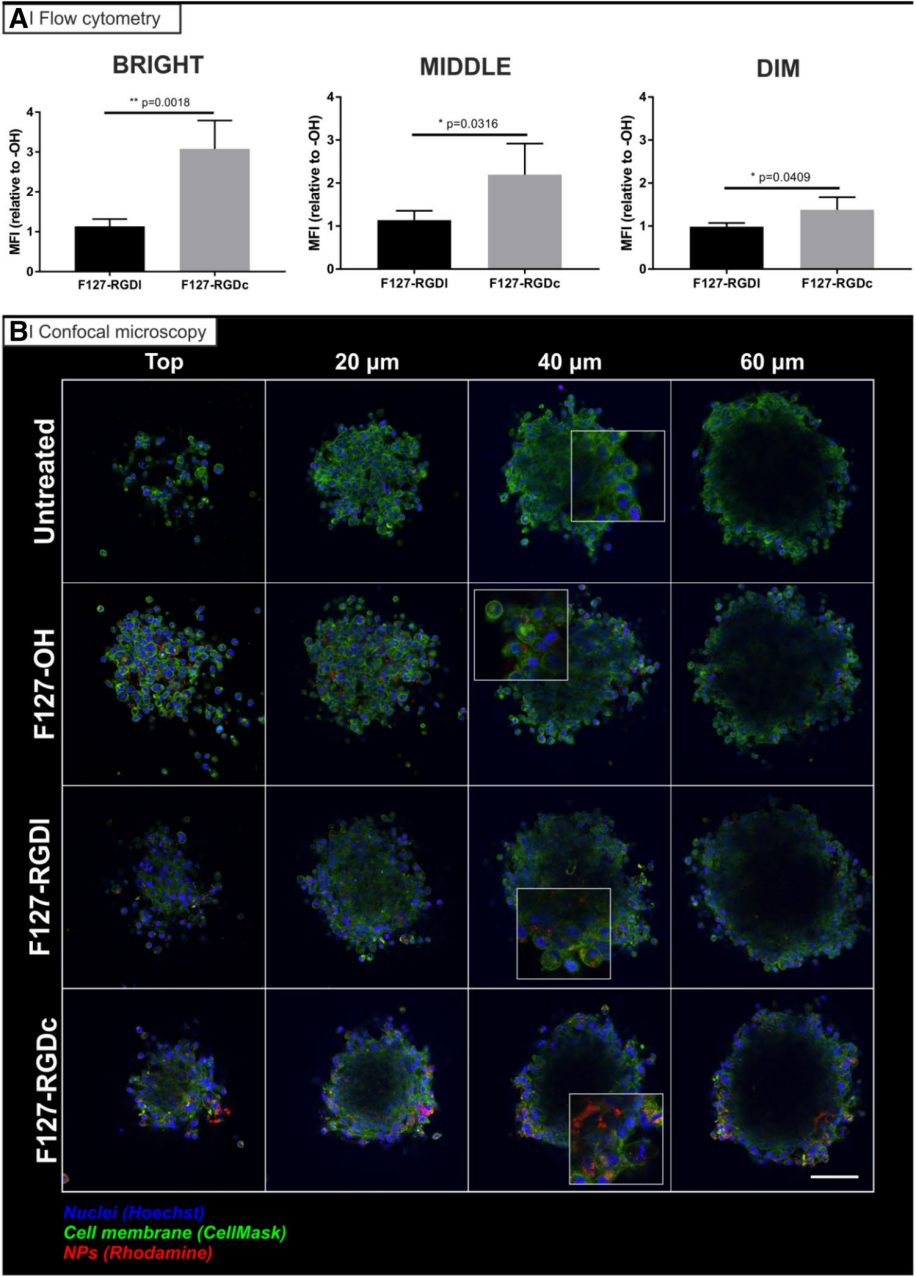
Penetration of rhodamine-labelled PLGA nanoparticles (red) prepared from Pluronic F127-OH, Pluronic F127-RGDl or Pluronic F127-RGDc (0 and 100% RGD/OH mixtures) in U87MG multicellular spheroids. (**A**) Flow cytometry analysis: spheroids of about 400 µm in diameter were treated for 24 h with the three different nanoparticle formulations in cell culture media (0.5 mg/mL) at 37 ˚C, and then incubated with Hoechst (to differentiate between the edge and the inner part of the spheroid) for 10 min before disaggregation with trypsin/EDTA and flow cytometry processing. The graphs are representative of 3 independent experiments. Statistical analysis (Unpaired t-test), n = 3. (**B**) Confocal microscopy analysis: spheroids of about 250 µm in diameter were treated for 24 h with the three different nanoparticle formulations in cell culture media (0.5 mg/mL) at 37 ˚C. They were then incubated with Hoechst for 10 min before confocal microscopy analysis. Images are representative of 3 independent experiments. Nuclei: Hoechst stain (blue); Cell membranes: CellMask (green). Scale bar = 50 μm.

The results can be summarized as follows:

1. Nanoparticle uptake was always energy-dependent. A first uptake control was carried out at 4 °C, and at that temperature a partial inhibition was recorded for all particles (between 30–50%), more significantly for non-targeted ones.
2. The entry mechanism of RGDc-decorated nanoparticles was both RGDc- and α_v_β_3_-dependent. Either an excess RGDc in solution or an α_v_β_3_ blocking antibody inhibited approximately 40–50% of their uptake (no effect of RDGl or α_5_β_1_-blocking antibody). These results match those reported by others for nanoparticles exposing cyclic RGD peptides on their surface^23^.
3. The entry mechanism of RGDc-decorated nanoparticles was scarcely α_5_β_1_-dependent, although still possibly involving integrins, and thereby supporting the above hypothesis of low affinity for α_5_β_1_. Their cellular uptake was not inhibited by incubation with either a α_5_β_1_- or α_5_β_1_-blocking antibodies, but was reduced with soluble RGDl, which suggests an uptake governed by other integrin receptors, rather than by the most abundant integrins, i.e. α_v_β_3_ and α_5_β_1_.
4. Non-targeted particles likely internalize through a macropinocytosis-dependent pathway, and this may be active also for RGDl-decorated ones. The use of EIPA (amiloride, common macropinocytosis inhibitor) produced a strong inhibition (about 60%) of the former and a much weaker inhibition (about 20%) of the latter. These results were further confirmed by confocal microscopy, using dextran as a macropinocytosis probe (Fig. 5B).
5. The uptake of particles may involve integrins also in the absence of RGD peptides: the α_v_β_3_-blocking antibody reduced the uptake also of the non-targeted particles (~ 20 to 30%). We currently lack a sound explanation of this effect, but one may elaborate on the macropinocytic internalization of these particles (point 4); the entry of a number of viruses via membrane ruffling followed by micropinocytosis has been shown to be mediated by α_v_β_3_^58^, therefore it may be possible to speculate that—in the absence of other more specific binding events—U87MG macropinocytosis may involve α_v_β_3_.

The overall picture seems therefore to be that RGDl-decorated nanoparticles behave rather similarly (macropinocytic uptake with a similar kinetics) to non-targeted ones, although the two particles may differ in the involvement of receptors in this process (non-targeted showing signs of α_v_β_3_ involvement).

Finally, we have incubated U87MG cells with the nanoparticle formulations (0 and 100% RGD content) for 1, 4 or 24 h, followed by the addition of Lysotracker in cell culture media to label (endo)lysosomes. Results in Fig. 5A show that RGDc-decorated particles seem to have been taken up faster compared to RGDl-decorated or non-targeted counterparts, which again corroborates flow cytometry findings reported in Fig. 4 about the preferential uptake of RGDc formulations. After a 4 h exposure, a strong fluorescent signal was already observed for RGDc-decorated particles which effectively co-localized with lysosomes, while on the other hand it took up to 24 h for the other two formulations to reach similar levels of lysosomal co-localization. Therefore, these data confirm that specific targeting of α_v_β_3_ with a RGDc peptide does not only control the levels of uptake of a given nanocarrier, but importantly it also shifts the pathway through which the carrier is internalized, in our case from a preferential macropinocytic entry (for naked particles, also for RGDl) to a receptor-mediated (endo)lysosomal pathway (for RGDc-functionalized particles). This shift in pathway is relevant for drug delivery in that the processing of carriers via the late endosome/lysosome formation has been proven essential for the functional delivery of many biomolecules of interest once released in the cytosolic compartment, for example exogenous mRNA^59^.

### Nanoparticle penetration into U87MG glioma spheroids

The inability of drugs (and drug delivery systems) to penetrate and distribute throughout solid tumors remains one of the main limiting factors for successful cancer treatment^60,61^. Besides, the prediction of the in vivo behavior of a drug carrier proves extremely difficult given that all its physico-chemical properties, chiefly its size, overall charge and surface (bio)chemistry, can drastically affect its ability to penetrate and accumulate in solid tumors^62^. In the last part of this study, we have thus evaluated particle penetration into multicellular spheroids, a more complex 3D model that should shed further light on the role of integrin-facilitated drug delivery to solid tumors. We have used the U87MG cell line as model because of its high integrin expression. Since we have produced nanoparticles differing almost exclusively in the surface chemistry (i.e. presence of –OH, –RGDc or –RGDl groups), we assume any difference in their behavior correlates with their differential targeting capabilities. In order to produce glioma spheroids suitable for penetration studies, we seeded U87MG cells in ultra-low adherent plates for 10 days (i.e. until they reached 400 μm in diameter), incubated them with rhodamine labeled nanoparticles, and finally disaggregated them and analyzed by flow cytometry. Untreated cells were used as negative control for rhodamine autofluorescence and Hoechst was used to differentiate three distinct regions of the spheroid, namely the edge (Hoechst highly positive cells^+^, labelled as “bright” in Fig. 6A), the middle (Hoechst positive cells, labelled as “middle” in Fig. 6A) and the inner part of the spheroids (Hoechst negative cells, labelled as “dim” in Fig. 6A). Our results demonstrated no significant differences for RGDl-decorated or non-targeted particles, but an enhanced penetration and accumulation mediated by the RGDc peptide for cells isolated from the periphery of the spheroid (namely a threefold increase compared to RGDl or naked particles), but also from those deeper in the spheroid (namely a twofold and 1.5 fold increase at middle and inner sections, respectively). These data were further corroborated via confocal microscopy analysis of the intact spheroids (250 μm diameter), which qualitatively revealed a greater number of nanoparticles penetrating throughout the glioma spheroids, suggesting that the enhancement of nanoparticle penetration was possibly due to an improved transcytosis mediated by α_v_β_3_ heterodimers (Fig. 6B). Please note that because of the low degree of light penetration (a well-known drawback in the confocal microscopy of spheroids^63^), it was not possible to observe the cells in the core of the spheroid (deeper than 20 μm). Our experimental findings are consistent with the enhanced penetration and accumulation reported for other cRGD-decorated nanosystems in U87MG spheroids, such as c(RGDyK)-functionalized micellar particles^64^ and polymeric micelles^65,66^, as well as for PAMAM-cRGD dendrimer bioconjugates^67,68^.

## Conclusion

In this study we have evaluated the uptake and intracellular processing of PEGylated PLGA nanoparticles by means of their surface decoration with two integrin targeting RGD peptides: a cyclic derivative with preferential targeting for α_v_β_3_ integrin heterodimers and a linear derivative with preferential (but not exclusive) targeting capabilities for α_v_β_3_ and α_5_β_1_ heterodimers. We have produced a library of targeted nanoparticles varying in RGD peptide format and surface density, but otherwise having identical physico-chemical properties, to evaluate their interactions with A2780 (low integrin expression, α_5_β_1_ > α_v_β_3_) and U87MG (high integrin expression, α_v_β_3_ > α_5_β_1_) cancer cell lines. In this regard, particle decoration with increasing densities of RGDc (but not RGDl) led to an enhanced nanoparticle uptake in U87MG cell cultures linked to a α_v_β_3_ integrin specific active internalization pathway (as demonstrated by selective inhibition experiments), additionally increasing their localization to lysosomes (2D cell culture), as well as their ability to penetrate and accumulate into 3D multicellular spheroids. On the contrary, RGDl seemed to be internalized predominantly via a macropinocytic mechanism, leading to comparatively lower internalization and penetration. The importance of adequate integrin targeting in the design of nanoparticle formulations was also highlighted by the lack of selective accumulation of particles functionalized with either of the peptides in A2780 cells. These results encourage further research on the use of RGDc as an effective means of improving drug delivery into integrin overexpressing solid tumors, especially for modalities that require the (endo)lysosomal pathway for a functional cargo cytosolic release and functioning, e.g. mRNA delivery carriers.

## Supplementary information

Supplementary information

## Data Availability

Data are available from the corresponding authors upon reasonable request and with the permission of AstraZeneca.
