# Supplementary material for: Microfluidic-assisted preparation of RGD-decorated nanoparticles: exploring integrin-facilitated uptake in cancer cell lines
Source: Sci Rep. 2020 Sep 2;10:14505. doi: 10.1038/s41598-020-71396-x (PMC7468293; doi:10.1038/s41598-020-71396-x)
Supplement: Supplementary file 1 — Supplementary information [file 41598_2020_71396_MOESM1_ESM.docx]

**Microfluidic-assisted preparation of RGD-decorated nanoparticles: exploring integrin-facilitated uptake in cancer cell lines**

Julio M. Rios De La Rosa^1†^*, Alice Spadea^1^, Roberto Donno^1,2^, Enrique Lallana^1^, Yu Lu^1^, Sanyogitta Puri^3^, Patrick Caswell^4^, M. Jayne Lawrence^1^, Marianne Ashford^5^, and Nicola Tirelli^1,2^*

^1^ NorthWest Centre for Advanced Drug Delivery (NoWCADD), School of Health Sciences, University of Manchester, Oxford Road, Manchester, M13 9PT, United Kingdom

^2^ Laboratory for Polymers and Biomaterials, Fondazione Istituto Italiano di Tecnologia, 16163, Genova, Italy

^3^ Advanced Drug Delivery, Pharmaceutical Sciences, R & D, AstraZeneca, Cambridge, United Kingdom

^4^ Wellcome Trust Centre for Cell-Matrix Research, Faculty of Biology, Medicine and Health, University of Manchester, Manchester Academic Health Science Centre, Manchester, M13 9PT, United Kingdom

^5^ Advanced Drug Delivery, Pharmaceutical Sciences, R & D, AstraZeneca, Macclesfield, United Kingdom

^†^ Present address: Cambridge Enterprise Limited, University of Cambridge, The Hauser Forum, 3 Charles Babbage Road, Cambridge CB3 0GT, United Kingdom

*Corresponding author:

Email: [juliomanuel.riosdelarosa@manchester.ac.uk](mailto:juliomanuel.riosdelarosa@manchester.ac.uk)

Email: [nicola.tirelli@iit.it](mailto:nicola.tirelli@iit.it)

**Supplementary Information**

**1SI. TEM analysis**

Pluronic-coated PLGA particles (i.e. 100% Pluronic F127, 100% Pluronic-RGDl and 100% Pluronic-RGDc) were prepared as described in the main manuscript. TEM imaging was performed on a Tecnai 12 BioTWIN Transmission Electron Microscope (FEI). Prior to sample application, 400 mesh carbon coated grids were treated by glow discharging (Emitech K100X) to render them hydrophilic. Then, each nanoparticle sample was applied to the treated grids followed by gently washing with deionised water, and then application of 1% Uranyl acetate (UA) solution. Excess of UA solution was removed using filter paper before the grids were dried. The acceleration voltage was set at 80kV, and a Gatan Orius SC 1000 camera was used to capture the TEM images.


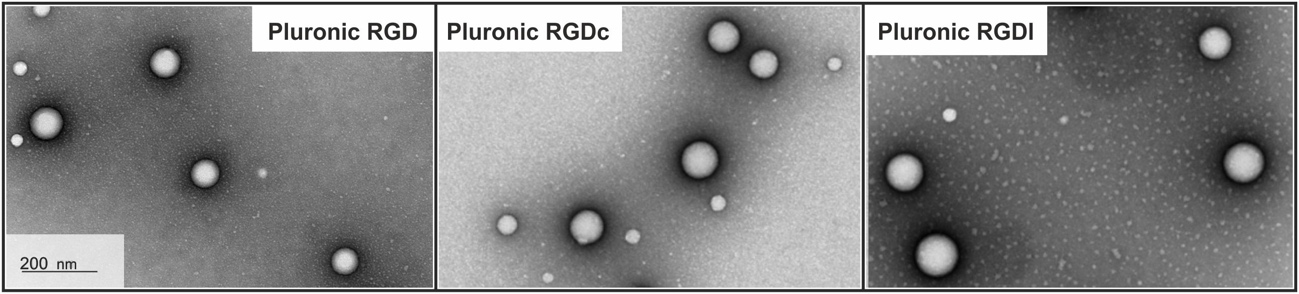


**Figure 1SI.** Negative staining TEM images of the nanoparticles; the average size of the nanoparticles in this dry state is around 80 nm, irrespective of the kind of Pluronic used for the surface coverage.

**2SI. Preparation of DiL- or Nile Red-loaded nanoparticles via microfluidics**

The automated microfluidic Asia 320 system (Syrris, Royston UK) was used for all preparations. A 0.015 % wt. surfactant aqueous solution (Pluronic F127) was mixed with a 0.31%wt. PLGA (RG502) acetone solution in an Asia 1000 μL 3-input reaction chip (Syrris part number: 2100146); for fluorophore-loaded particles, the acetone solution contained in addition to PLGA contained different amount of fluorophore: 0.02, 0.2, 2 and 20 µg/mL of DiL, or 0.05, 0.5, 5 and 50 µg/mL of Nile Red. The flow rates were controlled to have an acetone/water flow rate ratio of 0.2 and a total flow of 2 mL/min. Nanoparticles were then dialysed for 24h (Float-A-Lyzer with MWCO = 3500 Da were used) in order to replace acetone with distilled water.


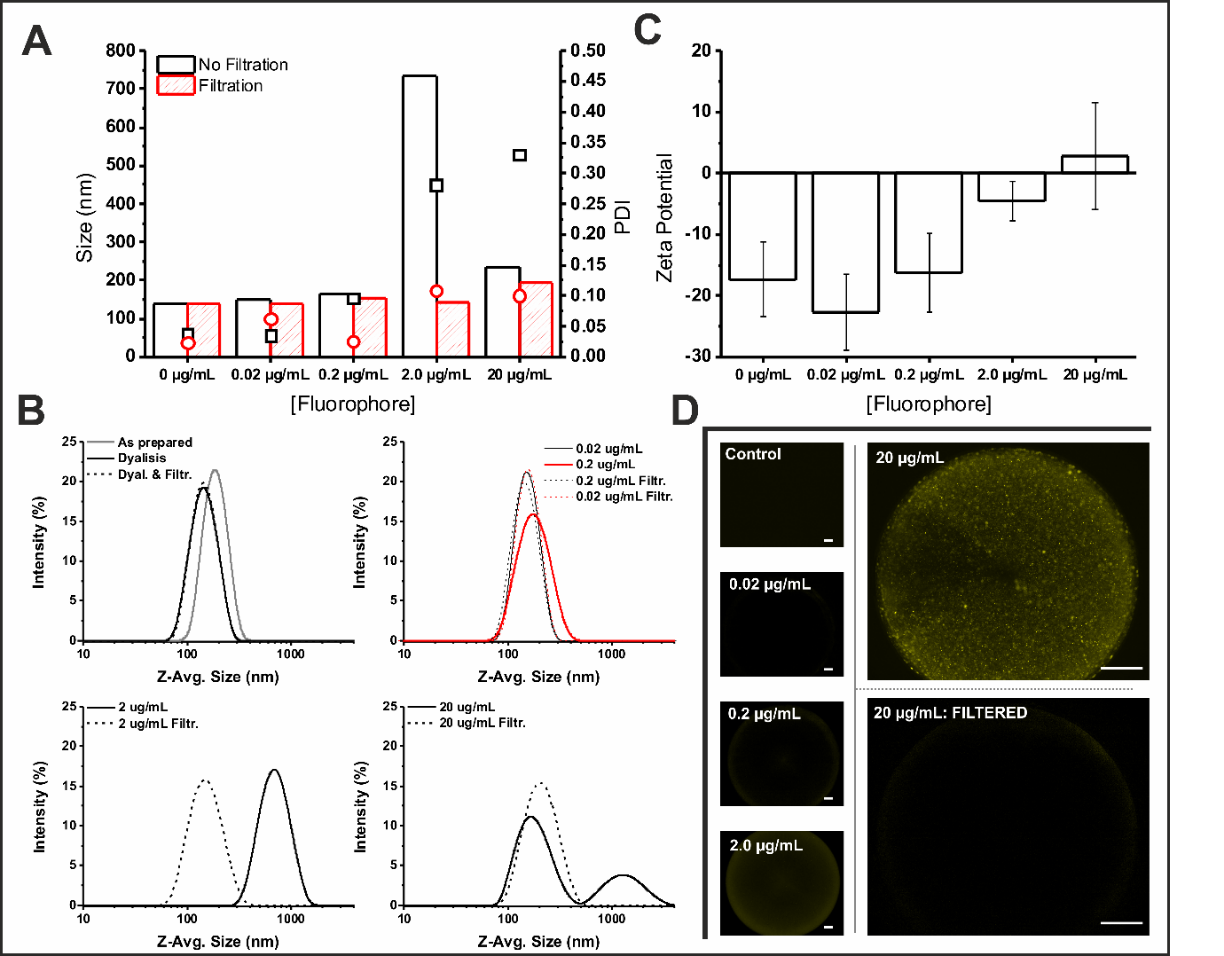


**Figure 2SI. DLS characterisation of Pluronic F12-stabilized PLGA nanoparticles loaded with DiL dye.** Increasing concentrations of DiL dye (i.e. 2 and 20 µg/mL) induced particle aggregation (A, B), most likely due to drastic changes in the particles surface charge (C) (n=3). **D.** Informative fluorescence microscope images highlighting the absence of detectable signal for low DiL loadings and the disappearance of the DiL signal upon filtration of the micron-size particle aggregates.


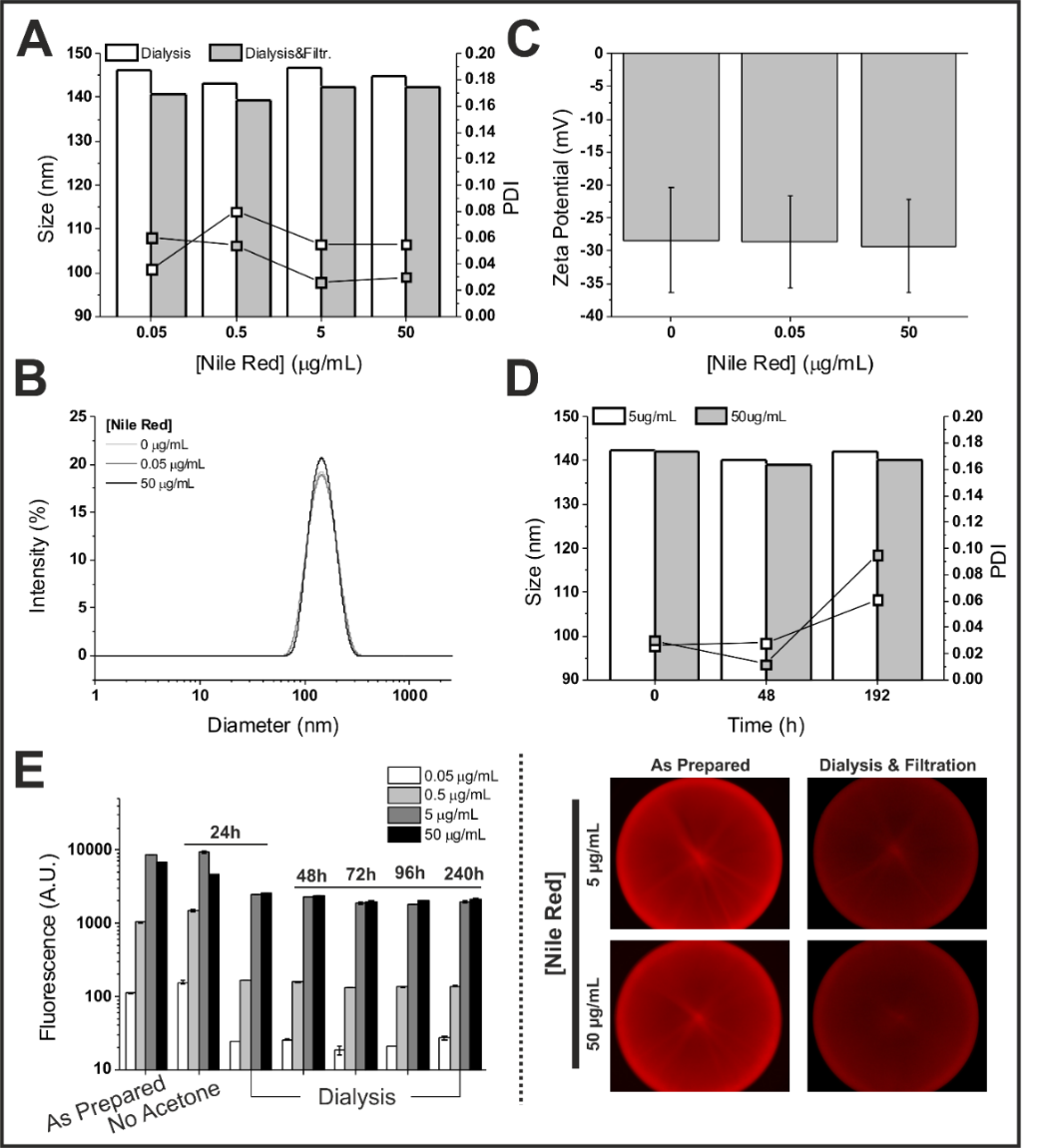


**Figure 3SI. DLS characterisation of Pluronic F127-stabilized PLGA nanoparticles loaded with Nile Red dye.** Nile Red dye was successfully encapsulated into PLGA nanoparticles with no significant effect on their size, PDI (**A, B**) or surface charge (**C**). The nanoparticle characteristics and fluorescence remain stable over time, even after dialysis (Float-A-Lyzer with MWCO=3,500 Da) and filtration (0.22 µm PES filters) (**D, E**) (n=3). **E.** Particle fluorescence could be successfully detected for high Nile Red loadings using a fluorescence microscope.


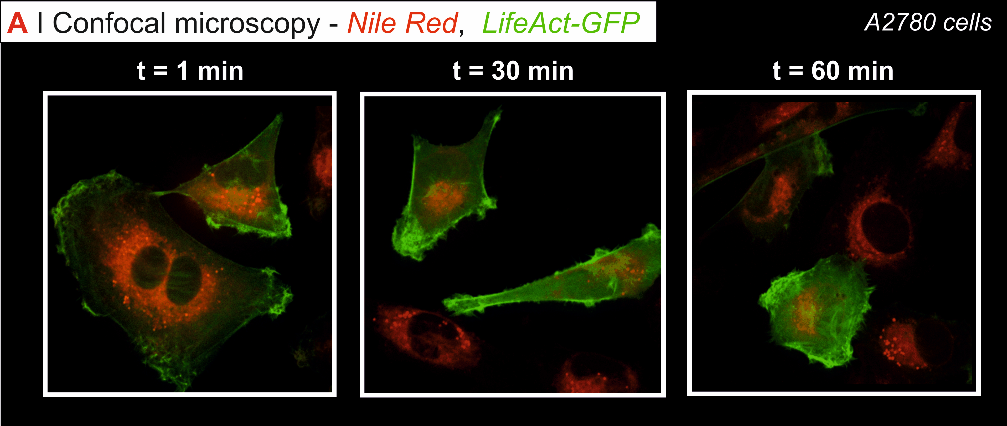


**Figure 4SI.** Representative confocal images of live A2780 ovarian carcinoma cells after exposure to Nile Red-loaded nanoparticles (0.5 mg/mL, RPMI medium supplemented with 10% FBS, T = 37ºC) over time. Actin was stained using LifeAct-GFP.

**3SI. Preparation of Rhodamine-labelled nanoparticles via microfluidics**


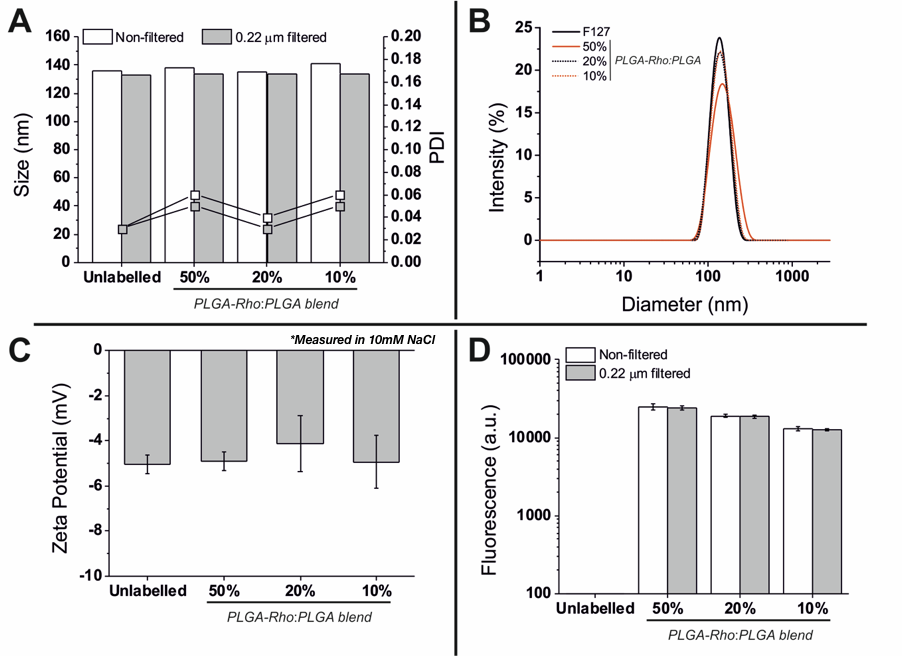


**Figure 5SI. DLS characterisation of Pluronic F127-stabilized PLGA nanoparticles prepared using PGLA labelled with Rhodamine B (PLGA-Rho).** The nanoparticle characteristics remained unaltered for all PLGA-Rho:PLGA blends under study (**A, B, and C**). However, fluorescence values were within the same order of magnitude for all formulations, and a 10% blend provided a high enough signal to accurately detect particles via fluorimetry (**D**). n=3.

**4SI. Selective Inhibition of Cell Uptake Pathways (U87MG cell line)**

Prior to flow cytometry experiments, the impact of uptake inhibitors/competitors on U87MG cell viability was assessed via MTS assay. Briefly, cells were seeded in Costar polystyrene 96-well plates with a flat bottom at a density of 1.5x10^4^ cells/cm^2^. Cells were left to adhere and grow until a confluency of ~70% was reached. Cells were then treated for 1.5 h at 37°C with growth medium containing either (i) EIPA 5 µM, (ii) 5 µg/mL α_v_β_3_ antibody, (iii) 5 µg/mL α_5_β_1_ antibody, (iv) excess RGDc peptide 2.5 µM, (v) excess RGDl peptide 2.5 µM, or (vi) PBS (negative control). Following treatment, cells were thoroughly washed with PBS and 120 µL of MTS solution in cell culture medium were added per well following manufacturer’s instructions. The absorbance at 490 nm was recorded after a 2 h incubation at 37 °C using a Synergy Biotek plate reader (equipped with Gen5 software). The read-outs were normalised to the untreated control (i.e. PBS-treated).


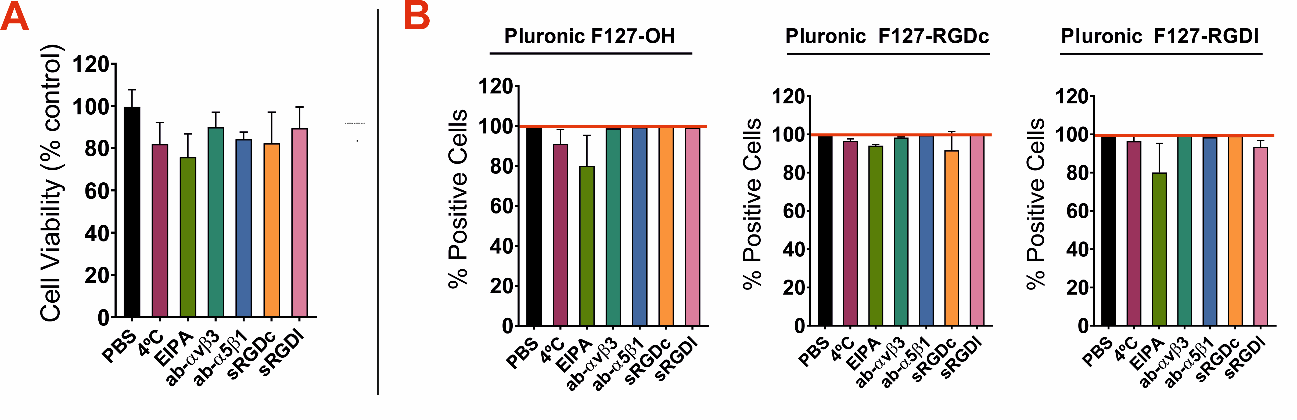


**Figure 6SI. (A**) Effect of uptake inhibitors/competitors on U87MG cell viability as determined via MTS analysis (mitochondrial reductase activity)**. (B)** Flow cytometry data for the blocking of PLGA nanoparticles internalisation. The percentage of positive cells as a function of the pre-treatment is presented here. n = 3.
